# Prevalence of *Dal* blood type and dog erythrocyte antigens (DEA) 1, 4, and 7 in canine blood donors in Italy and Spain

**DOI:** 10.1186/s12917-020-02351-1

**Published:** 2020-05-06

**Authors:** Daniela Proverbio, George Lubas, Eva Spada, Anyela Andrea Medina Valentin, Luis Miguel Viñals Florez, Maria del Rosario Perlado Chamizo, Roberta Perego, Maria Grazia Pennisi, Elisabetta Ferro, Luciana Baggiani, Alessandra Gavazza, Marie-Claude Blais

**Affiliations:** 1 Veterinary Transfusion Research Laboratory (REVLab), Department of Veterinary Medicine (DIMEVET), University of Milan, Via G. Celoria 10, 20133 Milan, Italy; 2 Veterinary Teaching Hospital, University of Pisa, Pisa, Italy; 3 Centro de Transfusion Veterinario (CTV), Arturo Soria 267, 28033 Madrid, Spain; 4 Laboratorio de Análisis Clínico, Hospital Clínico Veterinario, Universidad Alfonso X El Sabio, 28691, Madrid, Spain; 5 Department of Veterinary Sciences, University of Messina, Messina, Italy; 6 Department of Health, Animal Science and Food Safety (VESPA), University of Milan, Milan, Italy; 7 School of Biosciences and Veterinary Medicine, University of Camerino, Matelica, MC Italy; 8 Department of Clinical Sciences, Faculty of Veterinary Medicine, University of Montreal, Saint-Hyacinthe, QC Canada

**Keywords:** Blood group, Epidemiology, Transfusion reaction

## Abstract

**Background:**

The aim of this study was to determine the prevalence of Dal, and DEA 1, 4, 7 blood types, in a population of canine blood donors from Italy and Spain. Three hundred and twenty blood donor dogs receiving an annual health evaluation were included in the study. DEA 1 blood type was determined using an immunochromatographic strip technique while Dal, DEA 4 and 7 blood types were determined with polyclonal antisera using agglutination on gel columns.

**Results:**

Out of 320 dogs blood typed 7 (2 Cane Corso and 5 Doberman Pinschers) (2.2%) were Dal negative; 137 (42.8%) were positive for DEA 1; 320 (100%) were positive for DEA 4 and 43 (13.4%) were positive for DEA 7.

**Conclusion:**

This study showed a similar prevalence of DEA 1, 7 and 4 to that reported in previous studies in the same, and in different, geographic areas, and provides new data on the prevalence of the Dal blood group in Italy and Spain. There was no significant difference (*P* = 0.8409) between prevalence of Dal negative blood types found in our population (2.2%) and the prevalence reported in a canine blood donor population from the USA (2.5%). Our study identified Dal negative dogs in a previously tested breed i.e. Doberman Pinschers, but also the Cane Corso breed was found to have Dal negative dogs.

## Background

Blood groups are species-specific genetic markers on the surface of erythrocyte cell membranes which are defined according to antigenic recognition [1]. In dogs, there are seven internationally recognized blood groups in the Dog Erythrocyte Antigen (DEA) classification (DEA 1, 3, 4, 5, 6, 7, 8) [2] and, since these blood groups are independent, more than one blood group can be co-expressed on the surface of canine red blood cells [3, 4]. The DEA 1 is the most studied antigen. Worldwide prevalence of DEA 1 positive dogs has been estimated to be around 50–65% [5–9]. DEA 1 blood type is the most clinically important blood group in terms of transfusion reactions. Following the first transfusion with DEA 1 positive RBCs, DEA 1 negative dogs produce anti-DEA 1 antibodies and are at risk of potentially fatal, acute hemolytic reactions when a second DEA 1 mismatched transfusion is administered [10].

DEA 4 is a high frequency antigen in the canine population [9, 11–13], but there has been only one documented occurrence of a DEA 4 positive transfusion to a DEA 4 negative dog which had previously been sensitized by a DEA 4 unit of blood leading to a severe hemolytic transfusion reaction [14].

The prevalence of DEA 7 positive blood type varies between 5 and 82% depending on geographical localization, breed investigated and there is also probably an effect of the different methodologies used in the studies [11, 15–18]. An additional blood type, named Dal, was first recognized more than a decade ago. The Dal antigen was initially described in an anemic Dal negative Dalmatian accidentally sensitized by a first transfusion from a Dal positive canine blood donor [4]. A high percentage of Dal negative Dalmatians and Doberman pinchers have been reported in North America, whereas there is a high prevalence of Dal positive individuals in blood donor dogs from other breeds [19]. Dal negative blood donors might be difficult to find and, since Dal positive donors are common, Dal negative dogs are at increased risk of being sensitized by a first blood transfusion and face a high risk of acute hemolytic transfusion reactions following subsequent blood transfusion [20], as previous studies have shown that dogs have no naturally occurring anti-Dal antibodies [4, 16]. Euler et al. (2016), investigated the prevalence of two new blood groups, Kai 1 and Kai 2, and found that most dogs in North America were Kai1+/Kai 2-. No relationship was found between the Kai 1 and 2 and the DEA or Dal blood group systems. Furthermore a study [21] in Korea showed that dogs had no naturally occurring anti-Kai 1 and Kai 2 alloantibodies. A further survey in Germany found similar proportions of positive and negative of DEA 1,4, Kai 1 and 2 and Dal blood types to those in North America [22].

To the author’s knowledge, the prevalence of the Dal blood group has been investigated in only one blood donor dog population in USA, and there is limited published information on the prevalence of DEA 4 and 7, probably due to the limited availability of blood typing reagents. The aim of this study was to determine the prevalence of DEA 1, 4 and 7 and Dal blood group antigens in a population of blood donor dogs from Italy and Spain.

## Methods

### Blood samples

In this study, surplus blood samples in ethylene-diamine tetra acetic acid (EDTA) from 320 clinically healthy canine blood donors from the Mediterranean area of Europe were used. No dog had a previous history of blood transfusion. Canine blood donors (> 23 kg, 2–8 years old) were recruited from 3 University blood banks from northern (University of Milan, Lombardy), central (University of Pisa, Tuscany) and southern (University of Messina, Sicily) Italy and from a private blood bank (Centro de Transfusion Veterinario, CTV) in Madrid, Spain. The blood was collected from canine blood donors as part of routine annual health monitoring, and owners gave consent for the use of excess blood after routine testing in further studies. The study was conducted in accordance with European legislation (2010/63/EU) and was approved by the local bioethical committee of the University of Milan (EC26/2018). Data on gender, age and origin (where dogs lived and blood was collected, i.e. Northern, Central, Southern Italy or Spain) were collected from each dog sampled. Blood typing was performed at the Veterinary Transfusion Research Laboratory (Laboratorio di Ricerca di Medicina Emotrasfusionale Veterinaria, REVLab), University of Milan, Milan, Italy. After blood collection the samples were immediately sent to the laboratory. They were kept refrigerated and blood type was assessed within 7 days of blood collection [23]. To avoid agglutination interference, prior to performing blood typing, all samples were macroscopically evaluated for autoagglutination as follows: a drop of whole blood and saline was placed on a slide. The slide was rotated transaxially and the presence of agglutination was evaluated within 2 min. Microscopic autoagglutination was evaluated by microscopic evaluation (× 40). If macro or micro agglutination was present, the samples were excluded from the study.

### Blood typing

The polyclonal anti-DEA 4 and 7 antisera used in this study were purchased from ABRI (Animal Blood Resources International, USA) and imported to the University of Milan with the authorization of the Italian Health Minister (protocol authorization No. 0024135–23/09/2015- DGSAF-COD_UO-P). Dal blood typing was performed using polyclonal anti-Dal antibody obtained from Dal sensitized research Beagles from the University of Montreal, used undiluted as previous described [17].

In all samples blood typing was performed as described below.

DEA 1 blood type was determined using an immunochromatographic strip technique using monoclonal antibody (Lab Test DEA 1, Alvedia, Limonest, France) following the manufacturer’s guidelines.

DEA 4 and DEA 7 blood types, and Dal blood type, were determined with polyclonal antiserum using agglutination on gel columns (ID-CARD NaCl enzyme test and cold agglutinins, DiaMed GmgH, Cressier FR, Switzerland) as previously described [16, 17].

For all three blood types, DEA 4, DEA 7 and Dal, all blood samples mixed with polyclonal antiserum, were centrifuged in a dedicated gel column card centrifuge (ID-Centrifuge 24 S, DiaMed GmbH, Cressier FR, Switzerland) at 80×g for 10 min. The gel column cards were evaluated for the presence and strength of agglutination. The gel cards were visually interpreted and agglutination reactions graded from 0 to 4 + in accordance with the manufacturer’s instructions as follows [24]: 0, negative, all RBCs were at the bottom of the column; 1+, very few RBC agglutinates dispersed in the lower part of the gel, with most RBCs at the bottom of the tube; 2+, all RBCs were agglutinated and dispersed in the gel; 3+, some RBC agglutinates were dispersed in the upper part of the gel and most of the RBCs formed a red line on the surface of the gel; and 4+, all RBCs formed a red line on top of the gel. Results were interpreted as negative if no, or just 1+, agglutination was present, whereas ≥2+ agglutination reactions were considered positive [16, 19]. Results were considered valid if the control column containing only the RBC suspension and saline, was negative with all RBCs on the bottom of the column.

### Prevalence and statistics of dal positive dogs

Prevalence of the DEAs and Dal positive blood types were calculated as the proportion of samples testing positive for the different DEA and Dal blood types. The Pearson’s chi-square test was used for statistical comparison of Dal prevalence in our dog population with the prevalence of Dal in the blood donor population previously typed for Dal. Statistical software (Medcalc Software, version 16.4.3) was used for all analyses and statistical significance was set at *P* < 0.05.

## Results

Of the 320 blood donor dogs, 201 were female and 119 male; 50 were Spanish Greyhounds (from Madrid, Spain), 49 were Cane Corsos (35 from Messina, southern Italy and 14 from Milan, northern Italy), 46 were Doberman Pinschers (39 from Milan, northern Italy and 7 from Pisa, central Italy), 39 were Golden Retrievers (from Milan northern Italy), 25 were Boxers (from Pisa, central Italy), 22 were Argentine Dogos (from Milan, northern Italy), 19 were Siberian Huskies (from Milan, northern Italy), 18 were Samoyeds (from Milan, northern Italy), 17 were Bernese Mountain dogs and 35 were other breeds (13 mongrels, 5 Rhodesian Ridgebacks, 6 Irish Wolfhounds, 5 Italian Spinone, 4 German Shepherds and 2 Akita Inus, (all from northern Italy). No samples showed autoagglutination.

Of the 320 dogs blood typed for DEA 1, 137/320 (42.8%) were positive and 183/320 (57.2%) were negative. All 320/320 (100%) blood samples tested were positive for DEA 4, 43/320 (13.4%) were positive for DEA 7 and 277/320 (86.6%) were negative. Of the 320 dogs blood typed for Dal, 7/320 (2.2%) were Dal negative, 2 Cane Corso (one from northern and one from southern Italy) and 5 Doberman Pinschers (all from central Italy). Total prevalence of DEA 1, DEA 7 positive and Dal positive and negative dogs in breeds represented by > 10 individuals is reported in Table 1.

**Table 1:** Number and percentage of DEA 1 and DEA 7 positive and of Dal positive and negative dogs in breeds represented by > 10 individuals. All dogs were DEA 4 positive

Breeds	Blood type			
	DEA 1+ n (%)	DEA 7 + n (%)	Dal + n (%)	Dal - n (%)
Spanish Greyhound	9/50 (19.6%)	2/50 (4.0%)	50/50 (100%)	0
Doberman Pinscher	9/46 (19.6%)	3/46 (6.5%)	41/46 (89.1%)	5/46 (10.9)
Cane Corso	17/49 (34.7%)	0/49 (−)	47/49 (95.9%)	2/49 (4.1%)
Golden Retriever	38/39 (97.5%)	0/39 (−)	39/39 (100%)	0
Boxer	1/25 (4.0%)	13/25 (52.0%)	25/25 (100%)	0
Argentine Dogo	0/22 (−)	12/22 (54.5%)	22/22 (100%)	0
Siberian Husky	9/19 (47.3%)	2/19 (10.5%)	19/19 (100%)	0
Samoyed	5/18 (27.7%)	5/18 (27.7%)	18/18 (100%)	0
Bernese Mountain dog	17/17 (100%)	1/17 (5.8%)	17/17 (100%)	0

All seven Dal negative blood donor dogs were also negative for DEA 1 and 7 and positive for DEA 4. There was no significant difference (*P* = 0.84091) between the prevalence of Dal negative blood types in our population of blood donor dogs (7/320; 2.2%) and the blood donor dog population previously analyzed in USA (6/245; 2.4%) [19]. The percentage of Dal negative Dobermann Pinschers typed in our study (10.9%) was significantly lower (*P* = 0.00235) than those found in the USA (42.4%) [19].

## Discussion

Blood typing prior to canine blood transfusions minimizes the risk of transfusion reaction due to blood type incompatibility. Information on the prevalence of different blood types in various breeds aids the selection of blood donors for inclusion in a blood donor program [2]. This study showed a similar prevalence of DEA 1, 7 and 4 to that reported in previous studies in the same, and in different, geographic areas [16, 17, 19, 22].

Canine blood groups have been reported to vary geographically and between breeds [11–13], and the recognition of new canine blood groups, such as Dal and Kai 1 and Kai 2 antigens, demands further epidemiological studies to provide new information about the prevalence of these blood groups [4, 25]. In the USA, Dalmatians and Doberman Pinschers were identified as breeds with a high prevalence of Dal negative dogs [19]. However, Dal negative Beagles, Shih Tzus and Lhasa Apsos were also identified [19].

In the present study, using gel column technology and polyclonal anti-Dal antibodies, seven Dal negative blood donor dogs were identified from Italy and Spain with no significant difference between this population and the prevalence of Dal negative blood donor dogs in the population previously analyzed in USA [19].

The scarcity of Dal negative dogs among blood donors raises a concern that Dal negative dogs receiving an incompatible transfusion risk being sensitized. The Dal blood group is not, as previously hypothesized, a high-frequency antigen such as DEA 4, but seems to be missing in some dogs within certain breeds. There are also some breeds where > 10% of the dogs are Dal negative [19]. The identification of breeds with a high prevalence of Dal negative subjects would be advantageous to improve guidelines for blood donor dogs recruitment, until a commercial test is developed for Dal blood typing during routine clinical testing.

The Dal group seems to have an autosomal dominant mode of inheritance similar to that previously identified in other canine blood groups [19]. Furthermore, Dal positive parents usually produce only Dal positive offspring, and Dal negative offspring are uncommon if both parents are Dal positive.

Although, only a small number of Doberman Pinschers were typed (*n* = 46) in our study compared to the study in the USA (*n* = 432), we confirmed a high percentage of Dal negative dogs in this breed, although numbers were significantly lower than those found in the study from the USA [19].

The Dal blood group in Doberman Pinschers may have clinical importance, given the high prevalence of von Willebrand disease (vWD) in this breed [26]. Affected dogs have a high risk of bleeding and may need multiple transfusions [27], increasing their risk of being sensitized to the Dal antigen, developing Dal alloantibodies and experiencing a transfusion reaction.

The risk of an acute transfusion reaction linked to the Dal group, would most likely be confined to dogs receiving multiple transfusions as previous studies have shown that dogs have no naturally occurring anti-Dal antibodies [4, 16].

As there is no relationship between the DEA blood types tested and Dal positivity [19, 22], and because Dal testing is not currently routinely available, the only way to evaluate transfusion compatibility for the Dal group is by cross-matching. Cross-matching must always be carried out in dogs receiving further transfusions 4 days after the first. Our study also identified Dal negative dogs in the Cane Corso breed. This finding is of particular interest because the Cane Corso dog is an ancient Italian large breed dog that makes a very good blood donor for a number of reasons: large size (> 25 kg bodyweight), good temperament, easy and readily accessible jugular veins and significant higher DEA 1 and DEA 7 negative prevalence than most of the canine populations previously surveyed [7, 9, 11]. Our study also confirms the high prevalence of DEA 1 and DEA 7 negative subjects in this breed.

In this study, we typed only a small number of blood donor dogs, and further studies are required to estimate the real prevalence of Dal negative dogs in Doberman Pinschers, Cane Corsos and other blood donor breeds in Europe. Furthermore, the findings in the Cane Corso breed, which is classified in the Molossoid group, might prompt testing of other Molossoid breeds for identification of those with a higher prevalence of Dal negative individuals. As relevant antisera are not commercially available, it was not possible to assay DEA 3, 5 and Kai 1 and Kai 2 blood types [21, 22, 25], so the relationship of these with the Dal blood type could not be investigated.

## Conclusions

This study showed a similar prevalence of DEA 1, 7 and 4 to that reported in previous studies in the same, and different, geographic areas, and provides new data on the prevalence of the Dal blood group, in Italy and Spain. This study identified Dal negative blood donor dogs in a previously tested breed i.e. Doberman Pinschers, but the Cane Corso breed was also found to contain Dal negative dogs.

## Data Availability

The data analyzed during the current study are available from the corresponding author on reasonable request.

## Abbreviations

DEA: Dog Erythrocyte Antigen
EDTA: Ethylene-diamine tetra acetic acid
Lab Test DEA 1: Immunochromatographic strip technique using monoclonal antibody
vWD: von Willebrand disease
